# Global geographic and genomic epidemiology analysis of carbapenem-resistant *Escherichia coli* carrying *bla*_NDM-9_

**DOI:** 10.1128/msphere.00704-25

**Published:** 2025-11-25

**Authors:** Jie Sheng, Hao Lan, Xinru Wang, Jiayao Yao, Yueyue Hu, Jingyi Guo, Longjie Zhou, Xinyan Tang, Haotian Xu, Yunsong Yu, Xi Li, Xinhong Han

**Affiliations:** 1 Hangzhou Normal University 26494, Hangzhou, Zhejiang, China; 2 Centre of Laboratory Medicine, Affiliated People's Hospital, Hangzhou Medical College, Zhejiang Provincial People’s Hospital 117839, Hangzhou, Zhejiang, China; 3 Department of Clinical Laboratory, Zhejiang Cancer Hospital, Hangzhou Institute of Medicine (HIM), Chinese Academy of Sciences 89680, Hangzhou, China; Universita degli Studi di Napoli Federico II, Naples, Italy

**Keywords:** carbapenem-resistant *Escherichia coli*, NDM-9, phylogeographic, phylogenetic

## Abstract

Carbapenem-resistant *Escherichia coli* (CREC), particularly strains producing New Delhi metallo-β-lactamase-9 (NDM-9), pose a growing threat as agents of nosocomial infections. Despite their emergence since 2013, a comprehensive global phylogeographic and genetic characterization of *bla*_NDM-9_-carrying CREC is lacking. Through 7 years (2018–2024) of surveillance of CREC strains in a tertiary hospital, we obtained seven *bla*_NDM-9_-carrying CREC. Antimicrobial susceptibility testing, conjugative transfer experiments, whole-genome sequencing (WGS), and fitness analysis were performed. Publicly available genomes of *bla*_NDM-9_-carrying CREC from NCBI (curated by July 2025) were integrated for global analysis. All seven *bla*_NDM-9_-carrying CREC exhibited resistance to most antimicrobials tested, except colistin. WGS revealed diverse *bla*_NDM-9_-carrying plasmid types (IncB/O/K/Z, IncHI2, IncFIB, and IncC) and sequence types of strains (ST156 predominant). Key mobile genetic elements IS*26* and IS*CR1* facilitated *bla*_NDM-9_ dissemination. Plasmid structural analysis traced the evolution of the IncB/O/K/Z plasmid, revealing potential intra-hospital persistence and spread. Carriage of *bla*_NDM-9_-carrying plasmid imposed a significant fitness cost. Global analysis (*n* = 203 isolates) demonstrated high genetic diversity (56 STs), with ST156 (20.1%) being the most prevalent. Spatially, isolates were concentrated in Asia (China: 85.2%). Primary isolation sources were humans (39.4%) and chickens (34.9%), with a notable shift toward human predominance since 2016. Our findings elucidate the critical role of specific mobile elements in transmission, highlight the significant burden in China, document a shift toward human-associated isolates, and identify ST156 as a globally prevalent lineage. We emphasized the necessity of intensified surveillance to track the dissemination of *bla*_NDM-9_-carrying CREC.

**IMPORTANCE:**

This study provides the first integrative geographic and genomic epidemiology analysis of *bla*_NDM-9_-carrying carbapenem-resistant *Escherichia coli* (CREC). Our 7-year surveillance and genomic analysis revealed critical insights into the genetic characteristics and transmission dynamics of CREC carrying *bla*_NDM-9_. The identification of mobile genetic elements, such as IS*26* and IS*CR1*, underscores their role in the horizontal transfer of resistance genes, facilitating the spread of *bla*_NDM-9_. Furthermore, given the high frequency of *bla*_NDM-9_-carrying CREC in China and its likelihood of spreading clonally in hospitals, there is an immediate need to intensify surveillance efforts. Adopting a One Health perspective, our study highlights the interconnected antimicrobial resistance risks spanning human, animal, and environmental health domains, advocating for strengthened global phylogeographic and phylogenetic surveillance alongside clinical interventions to curb the spread of these high-risk epidemic clones.

## INTRODUCTION

Carbapenem-resistant *Enterobacterales* (CRE) represent a critical and rapidly escalating global health threat, owing to the rapid worldwide dissemination of multidrug resistance. The carbapenem resistance in *Enterobacterales* is mainly mediated by the production of carbapenemases. Among these enzymes, the New Delhi metallo-β-lactamases (NDM) represent a vital class of carbapenemases that hydrolyze virtually all β-lactams and have been reported worldwide (1). To date, 75 types of NDM enzymes have been identified in the NCBI database since the first identification of NDM-1 in India in 2008 (2). NDM-9, first detected in China in 2013 in *Klebsiella pneumoniae* (3), is a particularly concerning variant characterized by a single amino acid substitution (E152K) compared to NDM-1. Critically, this alteration confers significantly greater hydrolytic activity against carbapenems (4), potentially leading to more profound therapeutic failure. Furthermore, the dissemination of NDM-9, driven by the successful clonal spread of *Enterobacterales* strains and frequent horizontal plasmid transfer of *bla*_NDM-9_, exacerbates carbapenem resistance (5).

Carbapenem-resistant *Escherichia coli* (CREC), a predominant member of the CRE, has been categorized as a critical priority among the WHO bacterial priority pathogens (6). It has emerged as a high-risk pathogen for hospital-acquired infections worldwide, with a high incidence of urinary, digestive, respiratory, and blood infections (7, 8), undoubtedly creating greater challenges for clinical treatment (6). Furthermore, *bla*_NDM_-carrying CREC was widely distributed across diverse reservoirs, including humans (9), animals (10), and environment sources (11), creating complex transmission pathways under the One Health framework. Despite the recognized clinical significance of *bla*_NDM_-carrying CREC, research specifically focusing on the *bla*_NDM-9_ variant remains disproportionately scarce. While large-scale epidemiological surveys occasionally include *bla*_NDM-9_-carrying CREC, these strains often play a peripheral role, and dedicated, in-depth genomic studies exploring their global transmission dynamics, evolutionary history, and genetic drivers are notably lacking.

Therefore, this study aims to provide a comprehensive global genomic epidemiology analysis of *bla*_NDM-9_-carrying CREC. Through 7 years of active surveillance in a Chinese tertiary hospital, we identified and characterized seven clinical *bla*_NDM-9_-carrying CREC. Comprehensive genomic analysis of these strains revealed key molecular characteristics and identified the mobile genetic elements facilitating resistance gene dissemination. Furthermore, by integrating these findings with global genomic data, we conducted the first large-scale phylogeographic and phylogenetic analysis of *bla*_NDM-9_-carrying CREC, elucidating their evolutionary trajectories and global transmission patterns.

## RESULTS

### Characteristics of the CREC strains

The seven *bla*_NDM-9_-carrying CREC in this study were isolated from blood, drainage fluid, bile, rectal swab, puncture fluid, and sputum samples of seven patients aged 21–81 years (Table 1). The isolates were predominantly from male patients (71.4%, 5/7) compared to females (28.6%, 2/7), with six strains derived from individuals aged ≥50 years and only one from a 21-year-old. Antimicrobial susceptibility testing (AST) revealed that these isolates were all resistant to meropenem, imipenem, ertapenem, cefepime, ceftazidime, ceftazidime-avibactam, and ciprofloxacin, but sensitive to colistin (Table 2). There were four plasmid types found, two types of plasmids (IncB/O/K/Z and IncHI2) were successfully conjugated to *E. coli* J53, except IncFIB(AP001918) and IncC. Genome analysis revealed that plasmids capable of conjugation possess a complete set of conjugation elements, including oriT, relaxase, T4SS, and T4CP, whereas those that failed to conjugate lacked one or more of these essential components. Notably, all transconjugants exhibited fourfold to eightfold reduced minimum inhibitory concentrations (MICs) for ciprofloxacin and onefold to twofold elevated MICs for tigecycline compared to their donor strains.

**TABLE 1:** Information of 7 *bla*_NDM-9_-carrying CREC^*a*^

Isolates	Species	Collect date	Age	Sex	Ward	Clinical diagnosis	Sample source	Clinical outcome
CREC3019	*Escherichia coli*	2018/8/18	69	Female	Hepatological Surgery	Hepatapostema	Blood	Improved
CREC3024	*Escherichia coli*	2019/7/1	79	Male	Hepatological Surgery	Appendicitis	Drainage fluid	Improved
CREC3060	*Escherichia coli*	2020/8/7	50	Male	General Surgery	Choledocholithiasis	Bile	Improved
CREC2504	*Escherichia coli*	2021/8/9	81	Male	Hematology	Acute myelogenous leukemia	Rectal swab	Improved
CREC1123	*Escherichia coli*	2019/11/4	56	Female	Urology	Bladder cancer	Puncture fluid	Improved
CREC6306	*Escherichia coli*	2021/9/24	21	Male	Hematology	Acute B lymphoblastic leukemia	Rectal swab	Improved
CRECTL20	*Escherichia coli*	2023/4/10	51	Male	ICU	Chronic kidney diseases	Sputum	Improved

^*a*^ ICU, intensive care unit.

**TABLE 2:** Antimicrobial susceptibility testing results of 7 *bla*_NDM-9_-carrying CREC clinical isolates and transconjugants^*b*^

Isolates	Minimum inhibitory concentrations (MICs, µg/mL)										Plasmid type
	MEM	IPM	ETP	AMK	FEP	CAZ	CZA^*a*^	CST	TGC	CIP	
Clinical isolates											
CREC3019	128	16	>128	4	>128	>128	>128	<0.125	1	32	IncB/O/K/Z(121 k)
CREC3024	32	8	128	2	128	>128	>128	2	0.5	1	IncB/O/K/Z(111 k)
CREC3060	64	8	>128	2	>128	>128	>128	<0.125	1	32	IncB/O/K/Z
CREC2504	32	8	128	4	>128	>128	>128	<0.125	1	32	IncHI2,IncHI2A
CREC1123	128	16	128	>128	>128	>128	>128	0.25	0.125	32	IncHI2,IncHI2A(187 k)
CREC6306	128	32	>128	>128	>128	>128	>128	1	1	32	IncFIB(AP001918) (107 k)
CRECTL20	128	16	>128	>128	>128	>128	>128	1	1	32	IncC(108 k)
Transconjugants											
J3019_NDM9	32	4	128	1	64	>128	>128	<0.125	2	<0.125	IncB/O/K/Z(121 k)
J3024_NDM9	64	4	128	1	64	>128	>128	4	2	<0.125	IncB/O/K/Z(111 k)
J3060_NDM9	32	8	128	1	64	>128	>128	<0.125	2	<0.125	IncB/O/K/Z
J2504_NDM9	64	8	>128	8	128	>128	>128	<0.125	2	<0.125	IncHI2,IncHI2A
J53	<0.125	0.25	<0.125	2	<0.125	0.25	0.25	0.25	1	<0.125	/^*c*^
AT25922	<0.125	<0.125	<0.125	2	<0.125	0.25	0.25	0.25	0.25	<0.125	/^*c*^

^*a*^ Avibactam was added at 4 μg/mL.

^*b*^ MEM, meropenem; IPM, imipenem; ETP, ertapenem; AMK, amikacin; FEP, cefepime; CAZ, ceftazidime; CZA, ceftazidime-avibactam; CST, polymyxin; TGC, tigecycline; CIP, ciprofloxacin.

^*c*^ "/" is used to denote "unknown".

### Whole-genome sequence and comparative analysis

**Fig 1:**
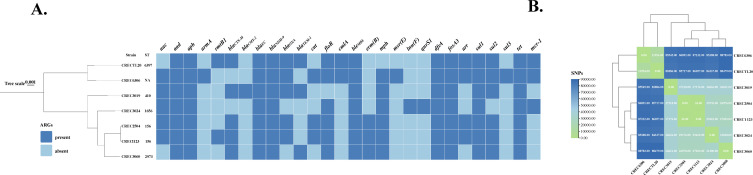
Characterization of the seven *bla*_NDM-9_-carrying CREC. (**A**) Phylogenetic analysis and basic information for the seven strains. (**B**) Cluster analysis of the seven strains based on single-nucleotide polymorphisms (SNPs). The SNP-based matrix obtained from Snippy was visualized using TBtools-II.

**Fig 2:**
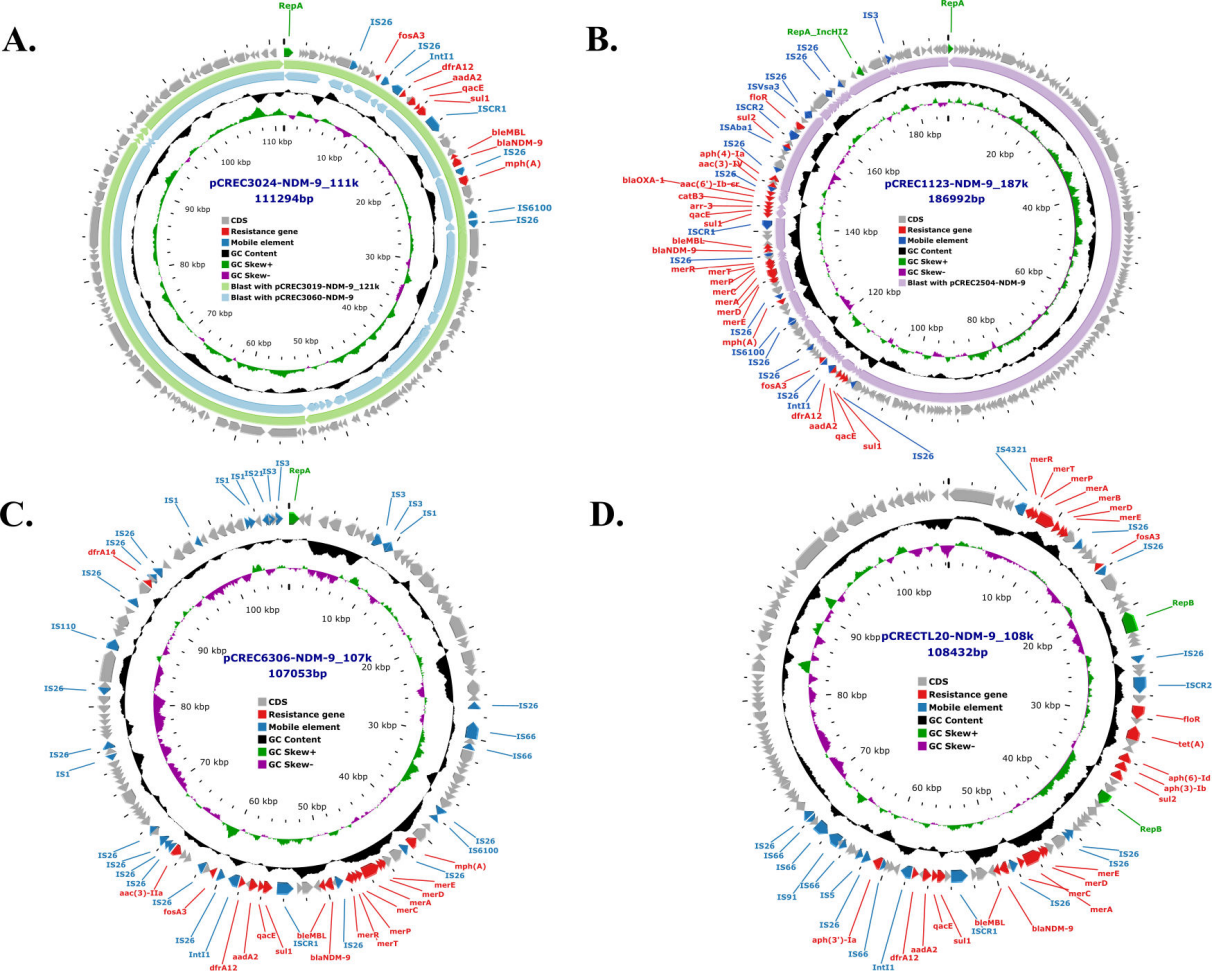
Circular sequence alignment of plasmids bearing *bla*_NDM-9_ in this study. (**A**) IncB/O/K/Z plasmid: pCREC3024-NDM-9_111 k blast with pCREC3019-NDM-9_121 k and pCREC3060-NDM-9. (**B**) IncHI2 plasmid: pCREC1123-NDM-9_187 k blast with pCREC2504-NDM-9. (**C**) IncFIB(AP001918) plasmid: pCREC6306-NDM-9_107 k. (**D**) IncC plasmid: pCRECTL20-NDM-9_108 k.

The five CREC isolates, based on third-generation sequencing, harbored a single chromosome (approximately 5 Mb) and carried two to six plasmids. Among them, *bla*_NDM-9_ was carried by one plasmid per strain. Two *bla*_NDM-9_-carrying CREC were subjected to second-generation sequencing. Various plasmids were identified in these seven CREC strains, including IncB/O/K/Z (*n* = 3), IncHI2 (*n* = 2), IncFIB(AP001918) (*n* = 1), and IncC (*n* = 1) (Table S1). All seven isolates also demonstrated significant diversity in sequence types (STs), with six different STs detected among the seven strains: ST156 (*n* = 2), ST410, ST1656, ST2973, ST6397, and unknown. WGS revealed that seven isolates with *bla*_NDM-9_ possessed 28 antibiotic resistance genes (ARGs) conferring resistance to 12 classes of antimicrobials: aminoglycosides (*aac, aad, aph, armA, rmtB1*), beta-lactams (*bla*_CTX-M_, *bla*_CMY-2_, *bla*_EC_, *bla*_NDM-9_, *bla*_OXA_, *bla*_TEM-1_), phenicols (*cat, floR, cmlA*), bleomycin (*ble*_MBL_), macrolides (*erm(B), mph, msr(E), lnu(F*)), quinolones (*qnrS1*), trimethoprim (*dfrA*), fosfomycin (*fosA3*), rifampicin (*arr*), sulfonamides (*sul1, sul2, sul3*), tetracycline (*tet*), and colistin (*mcr-1*). Notably, all seven strains harbored β-lactams (*bla*_NDM-9_), fosfomycin (*fosA3*), trimethoprims (*dfrA12*), aminoglycosides (*aadA2*), sulfonamides (*sul1*), and bleomycin (*ble*_MBL_) (Fig. 1A and 2A through D).

To delineate the evolutionary relationships between seven CREC-carrying *bla*_NDM-9_, we analyzed their core genomes using Snippy (Fig. 1B). Notably, the phylogenetic divergence among the seven strains was substantial, with single-nucleotide polymorphisms (SNPs) ranging from 0 to 88,783. Specifically, two strains of CREC6306 and CRECTL20 presented 11,954 SNPs, whereas interestingly, although CREC2504 and CREC1123 strains were isolated from different wards with a 1-year interval, they all belonged to ST156, with only 33 SNPs. As shown for SNPs, we observed that several isolates shared a few SNPs, suggesting possible clonal transmission (Fig. S1).

**Fig 3:**
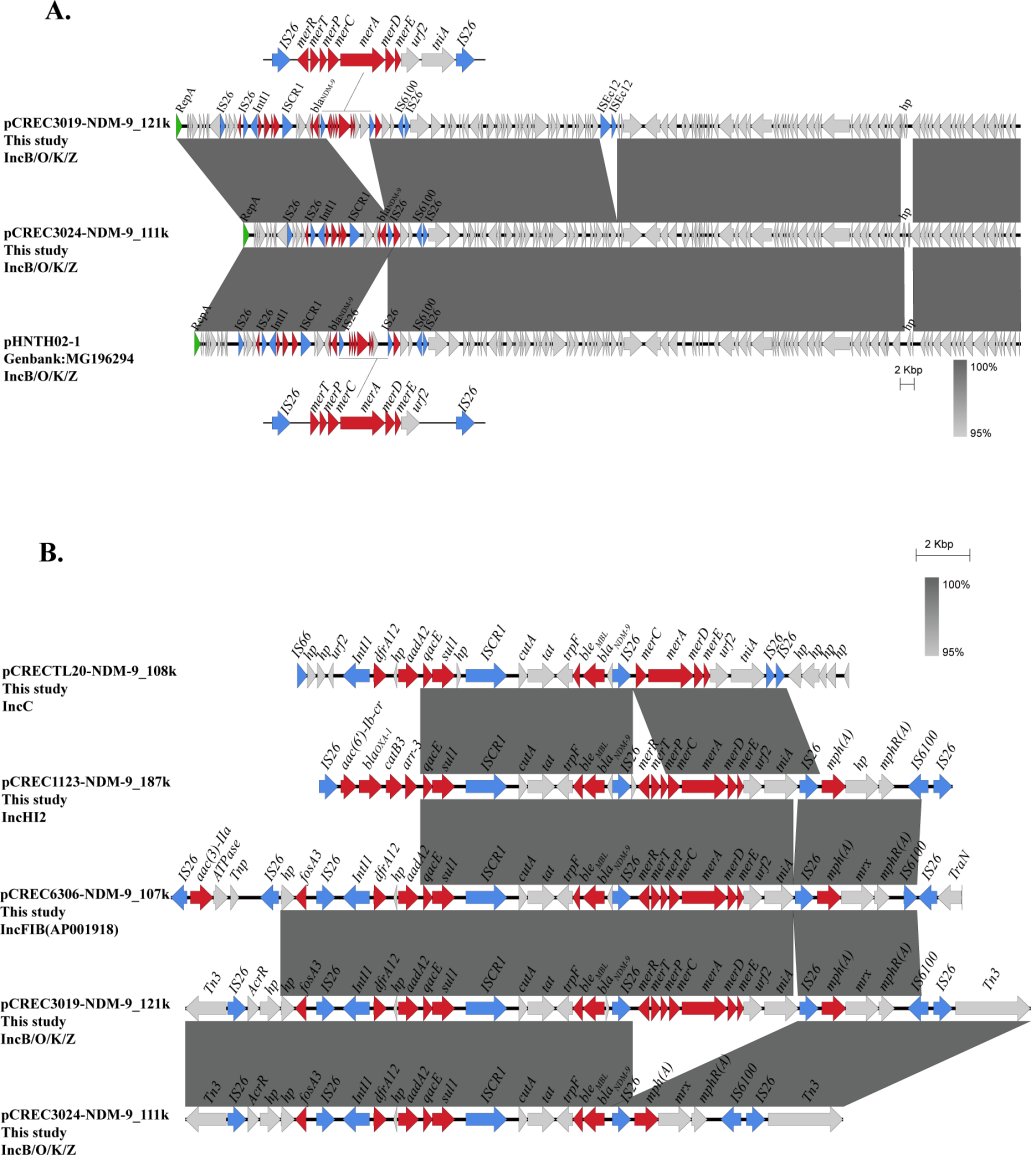
Alignment information via blast. (**A**) Clinical traceability of the IncB/O/K/Z plasmid acquisition of *bla*_NDM-9_ facilitated by IS*26*. Schematic comparison of structural features of pCREC3019-NDM-9_121 k, pCREC3024-NDM-9_111 k, and pHNTH02-1 via BLAST. (**B**) The core genetic environment for *bla*_NDM-9_-carrying CREC. Homologous regions (nucleotide identity of ≥95%) are highlighted with dark gray shading and squares. Arrows denote open reading frames and their transcription directions: red for genes encoding antibiotic resistance, blue for transposons and integron-related genes, green for genes involved in replication, and other genes are represented by light-gray arrows.

Observably, two isolates, CREC3019 and CREC3024, were collected from separate individuals in the same ward area of the same hospital, with an approximate 1-year interval between them, indicating that CREC3019 and others may function as long-term reservoirs for *bla*_NDM-9_-carrying IncB/O/K/Z plasmids (Table 1). This reservoir may serve as a conduit for intra-hospital spread, potentially culminating in the evolutionary process by which the IncB/O/K/Z plasmid acquired *bla*_NDM-9_ through a mobile element mediated by IS*26* (Fig. 3A). Second, sequence comparison of the plasmids revealed that pCREC3019-NDM-9_121 k and pCREC3024-NDM-9_111 k were highly similar. However, during the evolution, some fragments containing mercury resistance genes (IS*26-merR-merT-merP-merC-merA-merD-merE-urf2-tniA*-IS*26*), mediated by mobile elements IS*26*, were lost. Finally, a BLASTn search of GenBank showed that both pCREC3019-NDM-9_121 k (99.98% identity and 98% coverage) and pCREC3024-NDM-9_111 k (99.99% identity and 100% coverage) all matched highest with the *bla*_NDM-9_-carrying plasmid pHNTH02-1 (GenBank accession no. MG196294), which was previously recovered from *E. coli* THSJ02 from retail chicken meat in Guangzhou, China.

### Events associated with the spread of *bla*_NDM-9_

To further explore the core genetic environment of *bla*_NDM-9_ in CREC, we analyzed the *bla*_NDM-9_ region, which is approximately 20–30 kbp in length, through sequence alignment of the five plasmids from the seven strains in this study. The propagation of *bla*_NDM-9_ in a genetic context can be closely related to a common region (IS*CR1-cutA-tat-trpF-ble*_MBL_-*bla*_NDM-9_-IS*26*). Overall, the upstream and downstream regions of *bla*_NDM-9_ displayed notable variations mediated by IS*26*, IS*CR1*, IS*6100*, IS*66* elements, and the Tn*3* family of insertion and deletion sequences (Fig. 3B).

The hypervariable region of IncB/O/K/Z and IncFIB(AP001918) plasmids in this multidrug-resistant area consisted of five IS*26* elements flanking the following four different segments: (i) IS*26*-Δ*hp-fosA3*-IS*26*, (ii) IS*26-IntI1-dfrA12-aadA2-qacE-sul1* -IS*CR1-cutA-tat-trpF-ble*_MBL_-*bla*_NDM-9_-IS*26*, (iii) IS*26-merR-merT-merP-merC-merA-merD-merE-urf2-tniA*-IS*26,* and (iv) IS*26-mph(A)-mrx-mphR(A*)-IS*6100*-IS*26*. While in the upstream region of another plasmid IncHI2, a fragment (IS*26-aac(6’)Ib-cr-bla*_OXA-1_-*catB3-arr3-qacE-sul1*-IS*CR1*) is inserted. In the downstream region of the last plasmid IncC, lost (*merR-merT-merP,* IS*26-mph(A)-mrx-mphR(A*)-IS*6100*-IS*26*).

### Fitness effects of the *bla*_NDM-9_-carrying plasmids

**Fig 4:**
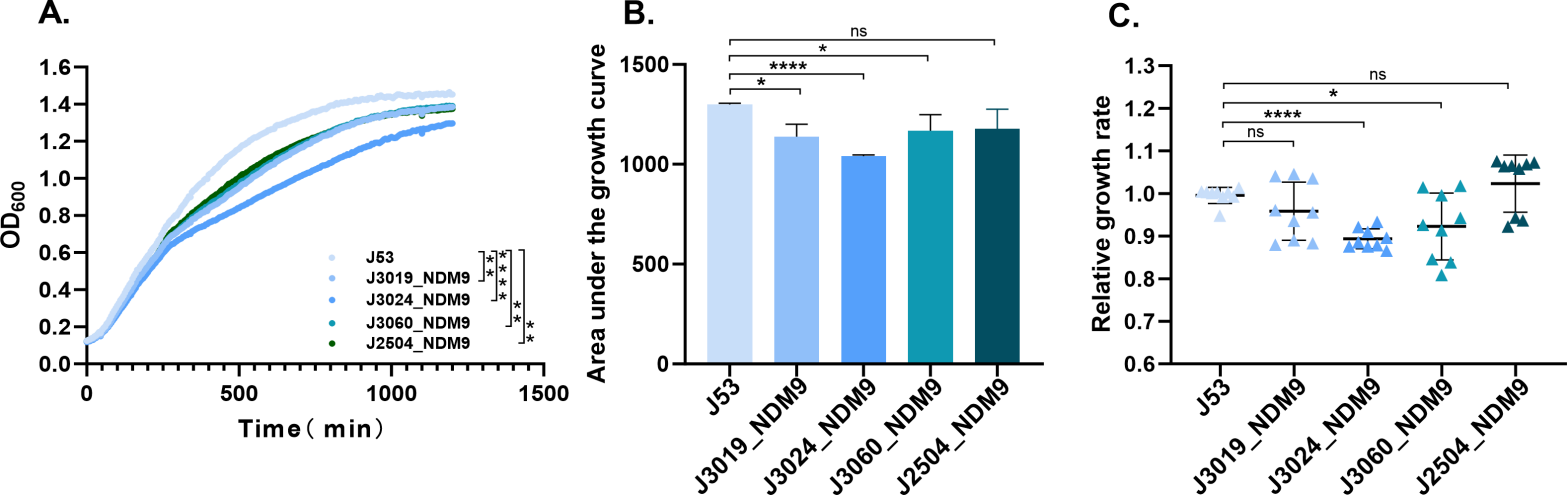
Growth profiles of the recipient strain *E. coli* J53 and transconjugants carrying *bla*_NDM-9_ across three measures: (**A**) Growth curve. (**B**) Area under the growth curve. (**C**) Relative growth rate. The graphed data reflect the average of three experiments, with the error bars representing the standard deviation. *P < 0.05; **P < 0.01; ****P < 0.0001; ns means *P* > 0.05 (ANOVA and two-sample unpaired *t*-test).

To estimate the fitness cost of the *bla*_NDM-9_-carrying plasmids, the growth curve, area under the curve (AUC), and relative growth rates between transconjugants and the recipient strain *E. coli* J53 were compared (Fig. 4A through C). Statistical analysis using unpaired *t-*tests revealed that *E. coli* J53 exhibited a significantly higher relative growth rate than the transconjugants. In particular, transconjugants harboring the IncB/O/K/Z plasmid (J3024_NDM9) demonstrated a pronounced growth disadvantage, with a mean relative growth rate of 0.8935 ± 0.0220  compared to 0.9956 ± 0.0179 for J53 (*P <* 0.0001). This fitness reduction was further corroborated by significantly lower OD_600_ values and reduced AUC in transconjugants. Collectively, these findings indicate that plasmid-mediated expression of *bla*_NDM-9_ imposes a measurable fitness burden on the host strain.

### Phylogeographic and phylogenetic analyses of *E. coli* with *bla*_NDM-9_

**Fig 5:**
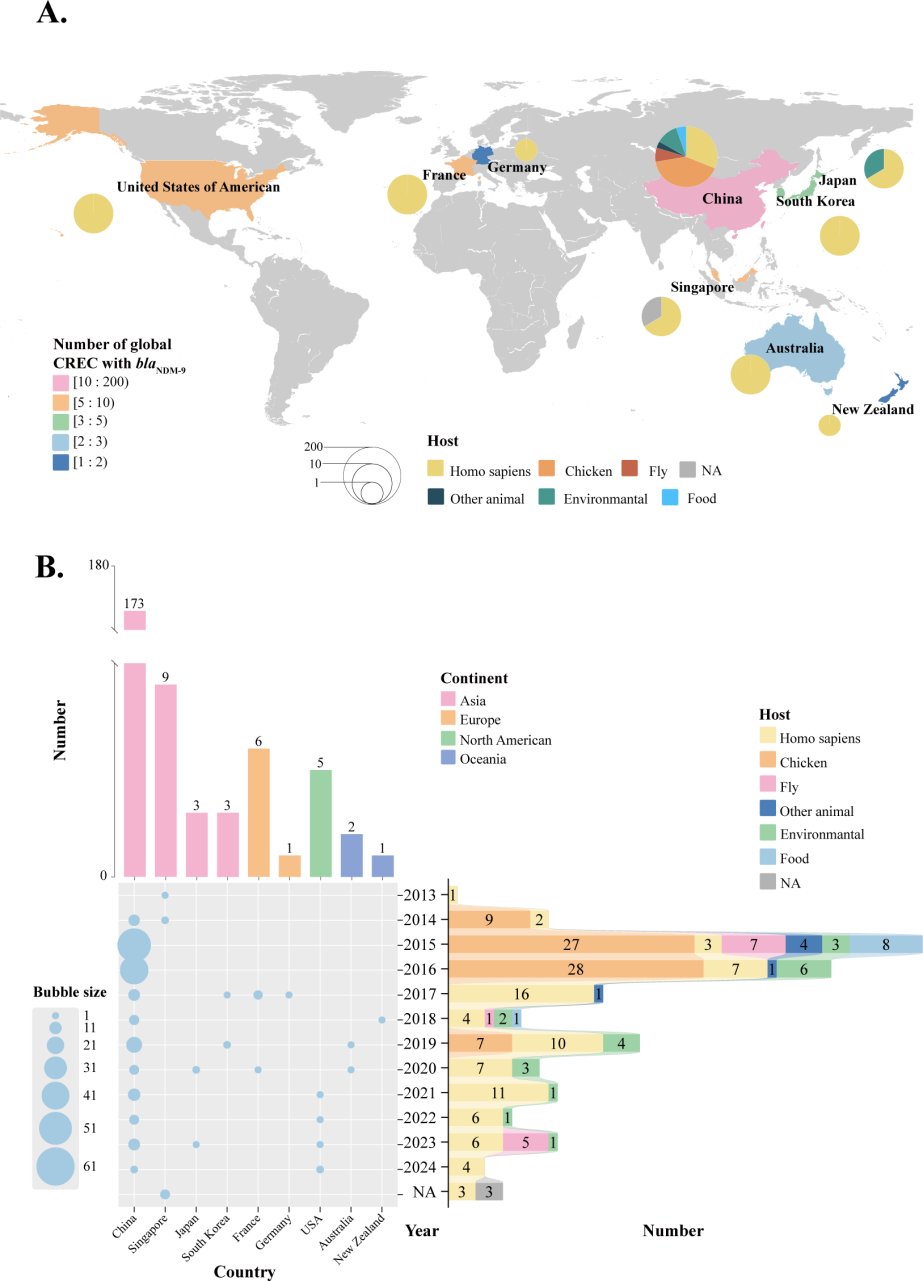
Global spatial-temporal distribution of 203 *bla*_NDM-9_-carrying CREC collected in this study and the NCBI database. (**A**) Global phylogeography of the 203 strains. Map lines delineate study areas and do not necessarily depict accepted national boundaries. (**B**) Timeline of the 203 strains. The bubble size was proportional to the number of strains. A bar chart illustrates the quantities and proportions of continent and host. NA: unavailable relevant information.

**Fig 6:**
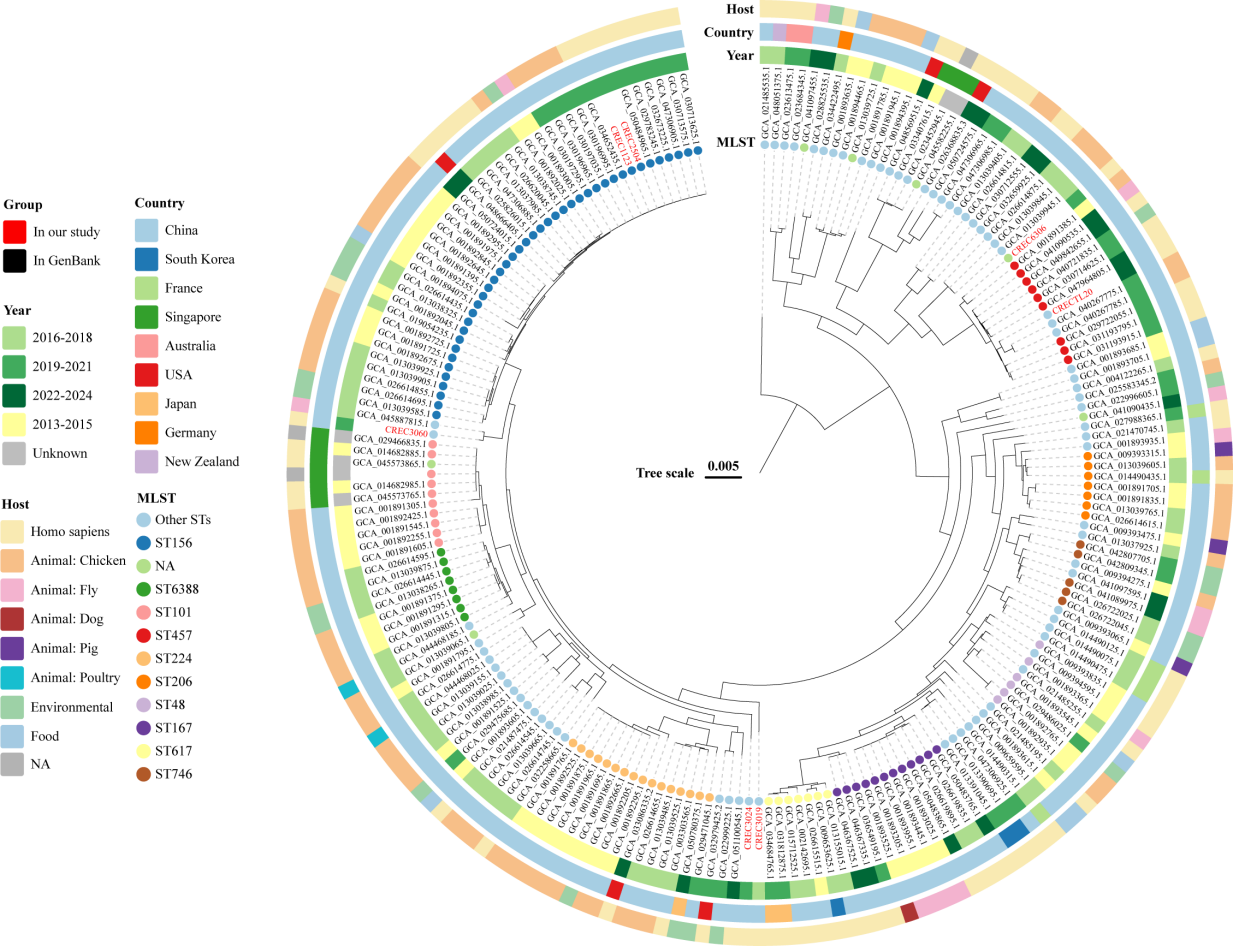
A phylogenetic tree of the 203 *bla*_NDM-9_-carrying CREC was obtained from this study and the NCBI database.

Phylogeographic and phylogenetic relationships among *bla*_NDM-9_-carrying CREC were investigated using 203 unique isolates retrieved from the NCBI database (Table S2). Geographically, *bla*_NDM-9_-carrying CREC was predominantly concentrated in Asia (China: 85.2%, 173/203; Singapore: 4.4%, 9/203; Japan: 1.4%, 3/203; South Korea: 1.4%, 3/203), followed by Europe (France: 2.9%, 6/203; Germany: 0.4%, 1/203), North America (USA: 2.4%, 5/203), and Oceania (Australia: 0.9%, 2/203; New Zealand: 0.4%, 1/203) (Fig. 5A and B). Multilocus sequence typing (MLST) analysis revealed 56 distinct STs, reflecting considerable genetic diversity. Notably, ST156 was the most prevalent, accounting for 20.1% (41/203) of the strains, followed by ST224 (7.8%, 16/203) and ST167 (5.9%, 12/203). Host association analysis revealed that human isolates accounted for 39.4% (80/203), followed by poultry (chicken: 34.9%, 71/203), environmental sources (10.3%, 21/203), flies (6.4%, 13/203), and food (4.4%, 9/203) (Fig. 6).

Genomic analysis identified a total of 135 virulence genes across the 203 *bla*_NDM-9_-carrying CREC, with 13 universally conserved genes (Table S3). These included iron-uptake genes (*entA, entB, entD, entE, entF, entS, fepB, fepC, fepD, fepG,* and *fes*) and outer membrane proteins (*csgG and ompA*). In addition, these 203 strains carried over 107 distinct ARGs (Table S4), with high prevalence of *ble*_MBL_ (100%, 203/203), *sul1* (99.0%, 201/203), *dfrA12* (90.6%, 184/203), *aadA2* (86.2%, 175/203), and *fosA3* (85.2%, 173/203). Notably, 49.8% (101/203) of strains carried more than 20 ARGs.

Temporal trends revealed a low global isolation rate of *bla*_NDM-9_-carrying CREC before 2015, followed by a gradual increase (Fig. 5B). Peaks in isolation occurred in 2015–2016, primarily in China, with subsequent dispersal to other regions. However, since then, the isolation rate has been scattered across countries. Host-source dynamics shifted markedly after 2016: while chicken sources initially dominated (pre-2016), human isolates subsequently surpassed them, suggesting evolving transmission pathways that warrant further investigation.

## DISCUSSION

In this study, we characterized seven *bla*_NDM-9_-carrying CREC isolated from clinical. Furthermore, we conducted a comprehensive analysis of the phylogenetic relationships of 203 *bla*_NDM-9_-carrying CREC, considering both geographical location and isolation source. Our findings provide a comprehensive depiction of them, thereby enhancing our understanding of these high-risk clones.

The prevalence of *bla*_NDM-9_-carrying CREC presents a great challenge for clinical treatment, not only because of its natural resistance to ampicillin (12) but also because of its acquired resistance to ceftazidime-avibactam, which is one of the last options for the treatment of carbapenem-resistant gram-negative bacteria (13). In our study, the seven CREC isolates were all resistant to third- and fourth-generation cephalosporins and carbapenems, which are commonly used clinically to treat infections caused by *Enterobacterales* (14). Fortunately, the AST results suggested that colistin was a viable option for treatment. However, this last-resort antibiotic has been challenged by the emergence of mobilized colistin resistance determinants (*mcr*) (15) and renal toxicity. Given the fact that it was urgently needed to research and develop some novel therapeutic alternatives (16).

WGS revealed the widespread presence of ARGs in the CREC strains. Plasmid diversity is the main reason for the spread of ARGs among bacteria (17). Most of these plasmids are transferable and pose a considerable risk of horizontal transmission between species. As shown by the SNPs, we observed that some CREC strains in our hospital currently exhibit a pattern of clonal dissemination. In addition, it is noteworthy that they all had a common core structure: IS*CR1-cutA-tat-trpF-ble*_MBL_- *bla*_NDM-9_-IS*26*. Moreover, IS*26* is classified as a pseudocomplex transposon. Previous studies have shown that IS*26* is involved in the spread and amplification of *bla*_NDM_ and may generate tandem repeat sequences through homologous recombination (18). Interestingly, two CREC isolates in our study were collected in the same ward from separate individuals within a year, illustrating the evolutionary process of the IncB/O/K/Z plasmid acquiring *bla*_NDM-9_ via a mobile sequence mediated by IS*26* and IS*CR1* over time, and the risk of plasmid dissemination in *Enterobacterales* in this hospital. These data strongly suggest that IS*26* and IS*CR1* are key factors in the emergence and rapid transmission of *bla*_NDM-9_. Simultaneously, IS*6100*, IS*66*, and Tn*3* elements seem to have also played an important role in the other ARGs transfer of *bla*_NDM-9_ isolates. Notably, these gene arrays, such as i–iv, were consistent with previous reports and were commonly identified in clinical and animal isolates (19–22). Our results further supported that these gene arrays may be associated with the transfer and spread of *bla*_NDM-9_-carrying plasmids. Additionally, our discovery of these plasmids supplements previous studies and further highlights the dissemination of *bla*_NDM-9_-carrying plasmids in human colonized isolates. These transmissions of resistance genes may confer species with “epidemic” clones (23). Therefore, it is necessary to establish a relevant nosocomial infection surveillance program to monitor the expansiveness and harmfulness of clinical mutant strains to prevent the emergence of high-risk epidemic clones (24).

Additionally, the fitness cost associated with the *bla*_NDM_-carrying plasmid is a critical factor influencing its dissemination and persistence in natural environments (25). In our growth rate determination experiments, the results also showed that the *bla*_NDM-9_-harboring plasmid, including IncB/O/K/Z and IncHI2, imposed a fitness cost on its bacterial host. The fitness cost observed may influence the plasmid’s long-term competition and persistence without antibiotics. However, constant clinical antimicrobial pressure likely counteracts this cost, enriching plasmid-carrying bacteria (26). Additionally, compensatory evolution in the host (27) or co-selection by other plasmid-borne resistance genes (28) may further stabilize the plasmid, promoting sustained *bla*_NDM-9_ dissemination among high-risk clones. Future studies should focus on experimental evolution models to unravel adaptive mechanisms. Furthermore, increasing evidence indicates that more types of *bla*_NDM_-carrying plasmids not only exhibit fitness cost (29), which facilitates their global prevalence (30, 31), but also mediate a multidrug-resistant phenotype. Hence, we speculate that this combination of traits may enhance their stability within bacterial hosts and facilitate horizontal gene transfer. Notably, given that only a few reports have addressed the adaptive costs of *bla*_NDM-9_-carrying plasmids, we have expanded upon previous investigations in this study.

Through phylogeographic analysis, we further discovered that Asia has emerged as the continent with the highest emergence of *bla*_NDM-9_-carrying CREC, especially in China, which was largely attributed to several multicenter studies (7). These observations implied that China could serve as a potential reservoir for *bla*_NDM-9_-carrying CREC. Furthermore, among the different host samples, the highest isolation rate was observed in human samples, followed by chicken samples. In recent years, the isolation rate in humans has been on the rise, indicating that the *bla*_NDM-9_-carrying CREC transitioned from an animal source to a human source, suggesting that they may have undergone interspecific transmission between animals and humans. This shift may be driven by intensified clinical screening (32), potential poultry industry regulations (10), global travel patterns (33), and the clonal expansion of human-adapted lineages like ST156 in healthcare settings (34). This indicates the increasingly significant role of *bla*_NDM-9_-carrying CREC in the spread and development of antimicrobial resistance. This trend is probably fueled by advancements in diagnostic methods, such as genomic sequencing (35), and more frequent human-animal contact, especially due to the consumption of animal-derived products (36). However, given that the sequencing resources are predominantly biased toward a few countries, and there is a lack of assessment regarding the epidemiological trends in some middle- to low-income countries, which needs further investigation to confirm these hypotheses and to elucidate the epidemiological dynamics of *bla*_NDM-9_ dissemination. Therefore, it is essential to widely adopt WGS and to conduct long-term global surveillance of *bla*_NDM-9_-carrying CREC to better understand its transmission dynamics and potential public health risks. The limited therapeutic options for high-risk clones like ST156 underscore the critical need for stringent infection control. Essential measures include active surveillance screening of high-risk patients, strict contact precautions and isolation for carriers, rigorous environmental decontamination, and robust antimicrobial stewardship to reduce selection pressure (37, 38). A bundled implementation of these strategies is vital to prevent the establishment and spread of these resistant lineages within healthcare settings.

## MATERIALS AND METHODS

### Clinical bacterial isolation and resistance gene screening

From 2018 to 2024, seven *bla*_NDM-9_-carrying CREC were collected from a tertiary hospital in China through active surveillance. Strain identification was performed using matrix-assisted laser desorption/ionization and time-of-flight mass spectrometry (MALDI-TOF MS; Sysmex-Biomeirers, France). The presence of carbapenemase genes was determined using polymerase chain reaction (PCR) and confirmed by Sanger sequencing, as previously described (39).

### Antimicrobial susceptibility testing

The MICs of 10 antimicrobial agents were determined, including meropenem, imipenem, ertapenem, amikacin, cefepime, ceftazidime, ceftazidime-avibactam, colistin, tigecycline, and ciprofloxacin, using the broth microdilution method with cation-adjusted Mueller-Hinton broth. The results were analyzed according to the 2025 CLSI guidelines (35th edition) (40), except for tigecycline, for which the European Committee on Antimicrobial Susceptibility Testing breakpoints were used (41). *E. coli* ATCC 25922 was used as the quality control.

### Conjugative transfer experiments

To investigate the transfer ability of *bla*_NDM-9_-carrying plasmids from *E. coli*, a conjugative transfer assay was performed using the recipient *E. coli* J53, as previously described (42). First, monoclonal colonies of donor and recipient bacteria were cultivated in 2 mL of Luria–Bertani (LB) broth, expanded to the logarithmic growth phase, mixed at a ratio of 1:1, and cultured on MH plates with a filter membrane (0.22 µm) overnight. Subsequently, transconjugants were screened on MH agar containing sodium azide (200 µg/mL) and ampicillin (100 µg/mL). Finally, the transconjugants were confirmed by PCR, MALDI-TOF MS, and AST determination.

### WGS and genomic analysis

WGS was conducted using the Illumina HiSeq and Nanopore MinION platforms at Zhejiang Tianke (Hangzhou, China), as previously described (43). The selection of strains for third-generation sequencing was strategically designed to capture the maximum plasmid diversity observed, while also in consideration of clinical relevance and temporal distribution. The only two that were not sequenced for the third generation, their plasmids can be matched with the mapping that has been completed, which means the types of these plasmids are consistent with those of the other sequenced strains. Complete genome sequences were assembled using the hybrid assembly tool Unicycler v0.4.8 (44) and annotated using the RAST (45). PlasmidFinder (46) from the Center of Genomic Epidemiology (https://www.genomicepidemiology.org/ services/) was used to identify plasmid types. MLST was performed using the MLST tool (https://github.com/tseemann/mlst). The virulence and ARGs of the strain were screened and identified using abricate (https://github.com/tseemann/abricate). BLAST from NCBI (https://blast.ncbi.nlm.nih.gov/Blast.cgi/) was used to identify similar sequences of plasmids and genes (47). Sequence comparisons were performed using BLASTn v2.4.0 (48) and visualized using Easyfig v2.2.3 (49) and Proksee (https://proksee.ca/) (50).

### Growth rate determination

Growth rate determination was used to evaluate the fitness cost of plasmids carrying *bla*_NDM-9_ (51). Three independent cultures per transconjugant were grown overnight in LB broth (with 200 µg/mL sodium azide and 100 µg/mL ampicillin), diluted 1:1,000 in LB broth without antibiotics, and aliquoted into a flat-bottom 96-well plate in triplicate. The plates were incubated at 37℃ with shaking at 200 rpm. OD_600_ curves were measured every 5 min for 20 h using a Bioscreen C MBR machine (Oy Growth Curves Ab Ltd., Finland). The relative growth rate was calculated using the R script based on the OD_600_ curves, and unpaired *t-*tests that returned a *P* value of <0.05 were considered significant.

### Global phylogeographic and phylogenetic analyses

To further monitor the global phylogeography and phylogenetic relationships among *bla*_NDM-9_-carrying CREC, by 15 July 2025, a public data set of the genomes (*n* = 223) was downloaded from the NCBI Genome Database (https://www.ncbi.nlm.nih.gov/pathogens) for our research. During the analysis, some duplicate and incomplete genomes were eliminated. Consequently, our study included 203 genomes, seven of which were obtained in this study. Species were delineated based on an average nucleotide identity (ANI) >95% using FastANI (https://github.com/ParBLiSS/FastANI) (Table S5) (52). Phylogenetic trees were built with FastTree (https://github.com/tseemann/snippy) (53) and visualized in ChiPlot (https://www.chiplot.online/) (54), while the global geographic distributions were mapped using ChiPlot and finalized in Adobe Illustrator v27.9.1. Heatmaps were utilized for the visualization of SNPs through TBtools-II (55).

## Data Availability

The complete sequences of the seven *bla*_NDM-9_-carrying CREC were submitted to GenBank, with the accession numbers listed in Table S1.

## SUPPLEMENTAL MATERIAL

Fig. S1 msphere.00704-25-s0001.pdf Cluster analysis of the 203 *bla*_NDM-9_-carrying CREC.

Supplemental tables msphere.00704-25-s0002.xlsx Tables S1 to S5.

ASM does not own the copyrights to Supplemental Material that may be linked to, or accessed through, an article. The authors have granted ASM a non-exclusive, world-wide license to publish the Supplemental Material files. Please contact the corresponding author directly for reuse.
