# Supplementary material for: Global geographic and genomic epidemiology analysis of carbapenem-resistant Escherichia coli carrying blaNDM-9
Source: mSphere. 2025 Nov 25;10(12):e00704-25. doi: 10.1128/msphere.00704-25 (PMC12724373; doi:10.1128/msphere.00704-25)
Supplement: Fig. S1 — Cluster analysis of the 203 blaNDM-9-carrying CREC. [file msphere.00704-25-s0001.pdf]

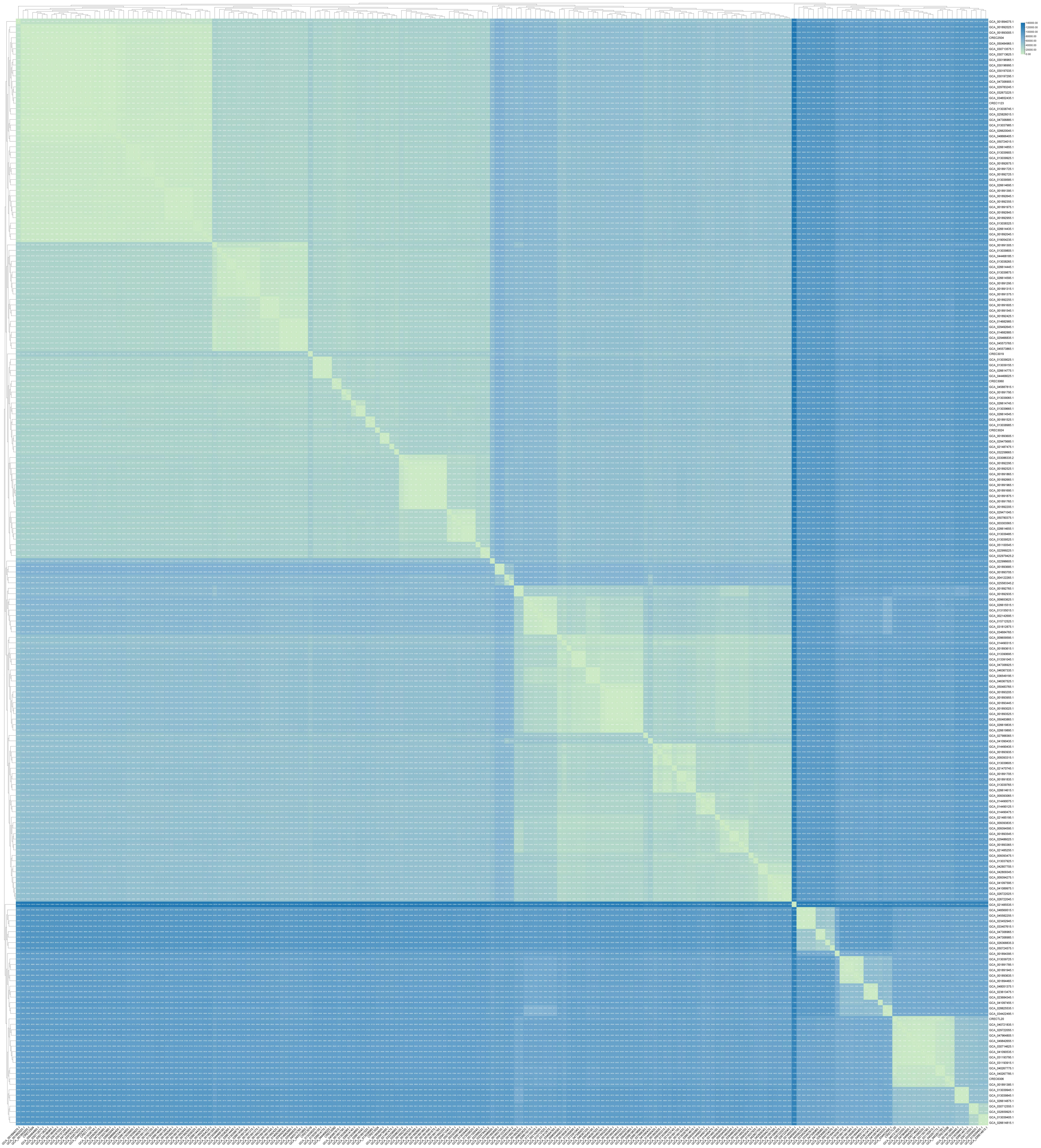

Figure S1. Cluster analysis of the 203 *bla*$_{\text{NDM-9}}$-carrying CREC based on single nucleotide polymorphisms (SNPs).
